# Management of rare biliary duct variants using a nasobiliary drainage tube and wire-guided navigation during laparoscopic cholecystectomy: a case report

**DOI:** 10.1055/a-2098-1439

**Published:** 2023-06-15

**Authors:** Cheng Zhang, Yun-sheng Suo, Sheng-long Zhang, Ke Sun, Yang He, Guang-kuo Li

**Affiliations:** Department of hepatobiliary surgery, Chengdu Second People’s Hospital, Chengdu, China

Biliary anatomical variation is an important risk factor for bile duct injury during laparoscopic cholecystectomy 1 . In the present case, we used a nasobiliary drainage tube and wire-guided navigation to identify a rare biliary variant and provide intraoperative guidance to reduce the risk of bile duct injury during laparoscopic cholecystectomy ( Video 1 ).

**Video 1** Use of a nasobiliary drainage tube and wire-guided navigation to identify a rare biliary variant and provide intraoperative guidance to reduce the risk of bile duct injury during laparoscopic cholecystectomy.


A 29-year-old woman was admitted with a 3-day history of upper abdominal pain and jaundice. Magnetic resonance cholangiopancreatography revealed that the cystic duct inserted into the right posterior sectoral duct (RPSD) and a suspected stone was located at the lower end of the common bile duct ( Fig. 1 ). Endoscopic retrograde cholangiopancreatography confirmed the presence of an abnormal junction between the cystic duct and RPSD ( Fig. 2 a ), classified as type 4A according to the right hepatic duct variant classification by Huang et al. 2 . Attempts to selectively cannulate the RPSD using a sphincterotome and guidewire were unsuccessful. However, we successfully cannulated the RPSD using a nasobiliary drainage tube (nasal biliary drainage catheter, ENBD-7-LIGUORY-C; Cook, Limerick, Ireland) and wire-guided navigation, confirming the aberrant junction of the cystic duct with the RPSD ( Fig. 2 b ). During surgery, the surgeon injected indocyanine green through the nasobiliary catheter to identify the RPSD and avoid its injury ( Fig. 3 ). Postoperative nasobiliary tube cholangiography revealed unobstructed flow in the RPSD ( Fig. 4 ).

**Fig. 1:**
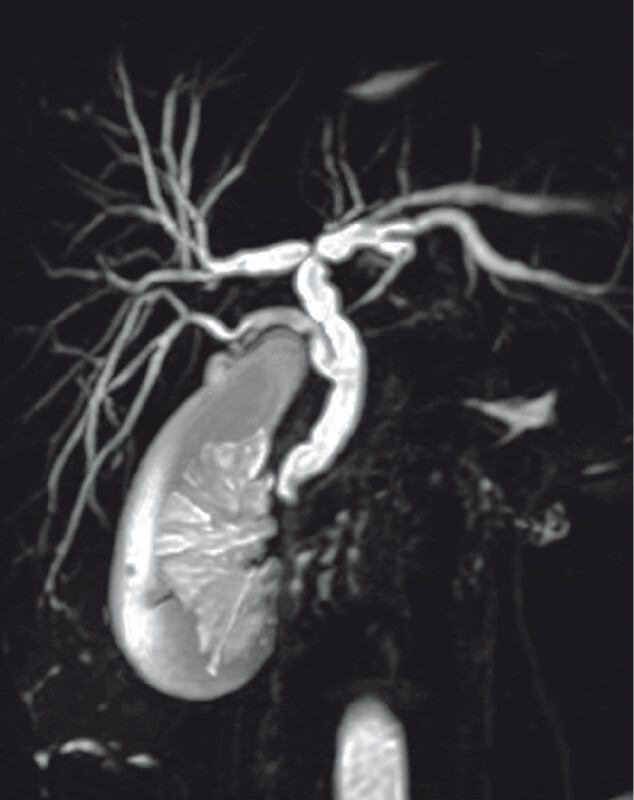
Magnetic resonance cholangiopancreatography (MRCP) showing that the cystic duct inserted into the right posterior sectoral duct and that a suspected stone was located at the lower end of the common bile duct.

**Fig. 2:**
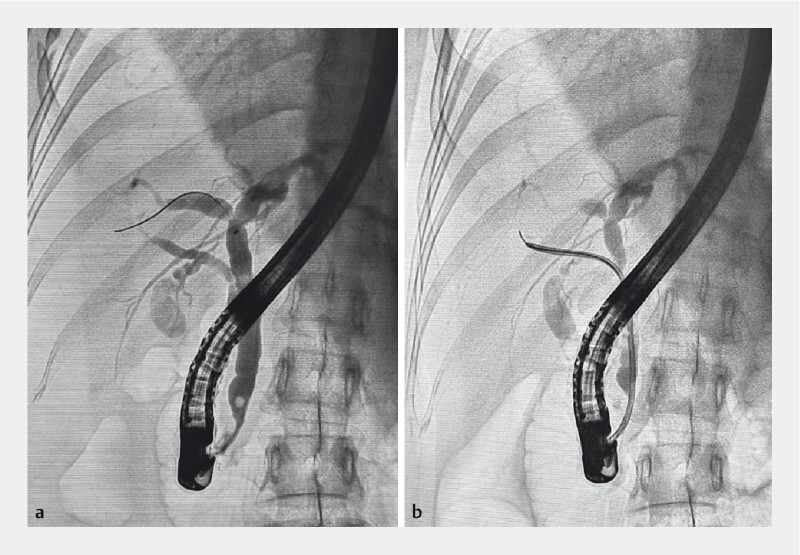
Endoscopic retrograde cholangiopancreatography showing: **a** a rare biliary anatomical variant; and **b** successful cannulation of the right posterior sectoral duct using a nasobiliary drainage tube and wire-guided navigation.

**Fig. 3:**
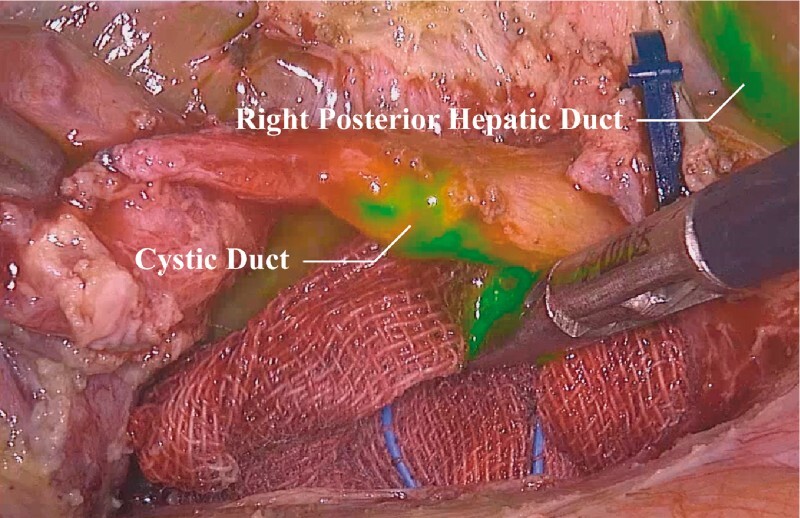
Injection of indocyanine green through the nasobiliary drainage tube to identify the right posterior sectoral duct and avoid injury to it.

**Fig. 4:**
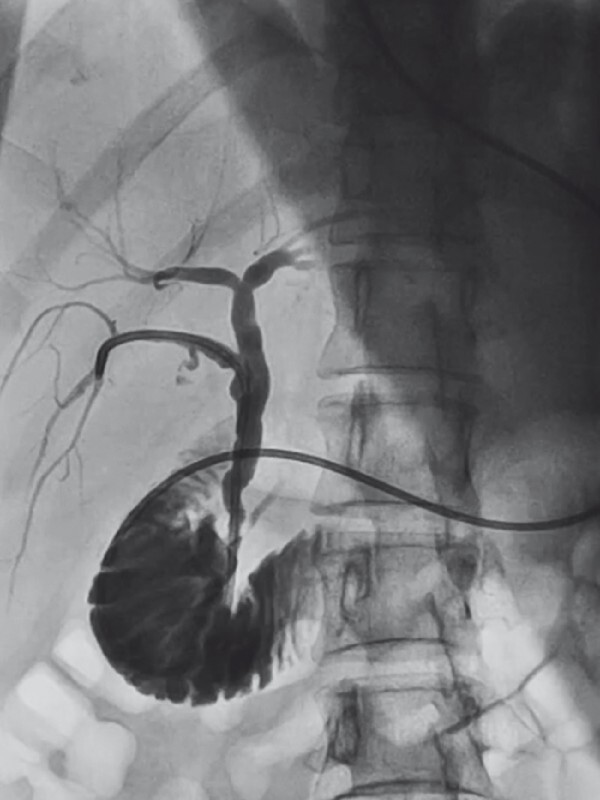
Postoperative nasobiliary tube cholangiography showing unobstructed flow in the right posterior sectoral duct.

Endoscopy_UCTN_Code_TTT_1AR_2AK

## Notes

Endoscopy E-Videos

https://eref.thieme.de/e-videos
*E-Videos* is an open access online section of the journal *Endoscopy* , reporting on interesting cases and new techniques in gastroenterological endoscopy. All papers include a high-quality video and are published with a Creative Commons CC-BY license. Endoscopy E-Videos qualify for HINARI discounts and waivers and eligibility is automatically checked during the submission process. We grant 100% waivers to articles whose corresponding authors are based in Group A countries and 50% waivers to those who are based in Group B countries as classified by Research4Life (see: https://www.research4life.org/access/eligibility/ ). This section has its own submission website at https://mc.manuscriptcentral.com/e-videos
